# Prevalence and Associated Factors of Depression in an Asian Community in Dar es Salaam, Tanzania

**DOI:** 10.1155/2018/9548471

**Published:** 2018-05-16

**Authors:** Sibtain M. Moledina, Khadija M. Bhimji, Karim P. Manji

**Affiliations:** ^1^Department of Internal Medicine, Muhimbili University of Health and Allied Sciences, Dar es Salaam, Tanzania; ^2^Department of Pediatrics and Child Health, Muhimbili University of Health and Allied Sciences, Dar es Salaam, Tanzania

## Abstract

Depression is a common condition in developed countries and is a growing problem in developing countries like Tanzania. Various risk factors have been identified through different studies. This study aimed at finding the prevalence of depression in a predominantly migrant Asian community and the behavioral, familial, social, and medical factors influencing it. A cross-sectional study among adults in a closed Asian community was done. Interviews and self-administered questionnaires were used to obtain details of symptoms and factors related to depression. DSM-IV criteria were used to diagnose depression in the individuals. Factors were assessed for significance using Chi square test. A total 384 participants were interviewed. Depression was found in 6.5% of the population. Risk factors included psychological stress (*p* < 0.001, OR = 6.37, 95% CI = 2.42–16.69) and a family history of depression (*p* = 0.023, OR = 2.57, 95% CI = 1.02–6.42). A sufficient family income was associated with a lower risk of depression (*p* = 0.013, OR = 0.21, 95% CI = 0.06–0.77). The prevalence of depression is within the range of the worldwide prevalence. Past psychological trauma and a family history of depression were significant risk factors, while a sufficient income was protective.

## 1. Introduction

Depression is a mental disorder characterized by feelings of depressed mood, loss of interest or pleasure in activities, and loss of energy that lasts for 2 weeks or more [1]. Everybody experiences mood changes daily. But when the depressive symptoms last longer over a period of days or weeks with changes in appetite and sleep pattern and disturbances in daily routine activities, a diagnosis of depression needs to be considered [1].

Depression occurs in people of all genders, ages, and backgrounds. It affects about 350 million people worldwide. Suicide is the worst complication of depression, with an estimated 800,000 people dying due to suicide every year [2].

Clinical interview is the best method for diagnosing depression [1]. Depression can be best diagnosed by the criteria set by DSM-IV or ICD-10.

Depression is associated with a number of risk factors like genetics; that is, it has been observed to run in families. Certain changes in neurotransmitter concentrations (particularly dopamine, serotonin, and norepinephrine) have been suggested to influence the occurrence of depression. Studies have shown that divorced or separated individuals and females are at higher risk. Life events such as early parental deprivation and social stressors also increase the risk of depression [3, 4].

It is estimated that, at a given time, 5–10% of the population are suffering from identifiable depression needing intervention.

Tanzania is one of the poorest countries in the world, with a gross national income of $300 per capita in 2003 [5]. The occurrence of mental health disorders is not uncommon in Tanzania. Ngoma et al. classified almost 24% of patients attending primary health clinics and 48% of patients attending traditional healers as having a common mental disorder by use of the Clinical Interview Schedule-Revised (CIS-R) tool [6]. In the same study, using the ICD-10 criteria, mild depression was found to be present in about 13% and moderate depression in about 2% of the study participants. Jenkins et al. studied common mental disorders among the general population and found an average prevalence of 2.5% [7].

There is a lack of literature on the use of DSM-IV criteria for diagnosing major depressive disorders. Furthermore, studies done in Tanzania have largely focused on the native population. There is a need to assess the migrant population, which constitute a minority that has been living in Tanzania for many years. Therefore, this study was aimed at determining the prevalence of depression and different risk factors for the diagnosis in an Asian community in Dar es Salaam, Tanzania.

## 2. Materials and Methods

A community-based, cross-sectional study was conducted where all adults above 18 years of age who consented were included. The population consisted of a migrant Asian community in Dar es Salaam (Tanzania), most of whom with an ancestral background leading to India or Pakistan. Based on the official website of the community [8], the initial ancestors migrated to Dar es Salaam in 1875 and established a center in 1908. This was then expanded in 1968 when the community grew in number after more people had migrated from India and Pakistan. Currently, the community is comprised of a population of 8,500 people. The entire community consisted of approximately 4500 adults, mostly born in Tanzania and now living in an urban setting. The sample size for the study was calculated by the use of the Kish-Leslie formula, with an estimated prevalence of 50% due to the lack of enough studies on depression in Tanzania. Patients were recruited consecutively as they arrived at the study site during a community event which is widely attended (over 90% attendance). This event was the annual health screening done in the community center for community members where basic screening for height, weight, blood pressure, and blood sugar is done. This screening was available for adults of the community and separate sections were kept for males and females. Members who attended the event were screened for the above and were then invited to participate in this study. None of the members refused to participate in the study. After the above basic screening was completed, participants were sent to another station where privacy was ensured and the questionnaire was administered by the research assistants.

Data was collected on the risk factors and DSM-IV criteria for depression via structured questionnaires. Interviews were conducted by the research assistants who were medical students with training in psychiatry and who also received initial training on taking interviews for this study. The questionnaires enquired about behavioral, social, familial, and medical factors which may influence the incidence of depression, in addition to having a checklist of the symptoms of depression as per DSM-IV criteria.

Permission to conduct the study was sought from the community medical board. Interviews with participants were kept confidential. Any participants found to have depression by the DSM-IV criteria were advised to seek further treatment with their primary physician or a specialist. Additionally, participants who were found to have stressors but not meeting the criteria from depression were also advised to seek help from their primary physician.

Prevalence of depression was determined using the DSM-IV criteria. Associations with the various factors were done using the *p* value (significance being *p* < 0.05). The strength of the association was measured using Odds Ratio and 95% Confidence Interval.

## 3. Results

A total of 384 individuals were interviewed, of whom 218 (56.8%) were males. Individuals who were above 44 years of age were the most interviewed participants in the study group, contributing 39.3% of the sample population. Other demographic parameters are shown in Table 1.

Using the DSM-IV criteria for diagnosing depression, 6.5% (25 out of 384) of the study population had depression. A descriptive analysis studying the risk factors for depression in the community was done and the results are shown in Table 2. Participants who had experienced a psychological traumatic event in the past had a higher proportion of depression (22.7%) than those who had no history of any such psychological trauma (4.4%). This was statistically significant (*p* < 0.001). A family history of depression was also significantly associated with a diagnosis of depression (*p* = 0.023), with 11.9% of patients with a positive family history having a diagnosis of depression, in contrast to 5.0% of depressed patients with no family history of depression. Other risk factors suggestive of contributing to a diagnosis of depression were female sex (*p* = 0.08) and smoking (*p* = 0.088).

Having a sufficient family income was shown to be significantly protective (*p* = 0.013) against depression. Out of 266 individuals who responded to the question about family income, there were four times more patients with depression who had no sufficient income than those who had a sufficient family income (20.0% to 5.0%, resp.).

Recurrent thoughts of death/suicide were more common among depressed individuals than among their nondepressed counterparts. Six of the depressed individuals (24.0%) were found to have recurrent thoughts of death/suicide compared to 2.8% of nondepressed individuals with this symptom. This difference was statistically significant (*p* < 0.001, OR = 11.02, 95% CI = 3.13–38.23).

Factors that did not seem to affect the prevalence of depression among the study group were age (*p* = 0.322), education level (*p* = 0.768), marital status (*p* = 0.433), presence of comorbidity (*p* = 0.129), and number of hours spent exercising per week (*p* = 0.495).

**Figure 1:**
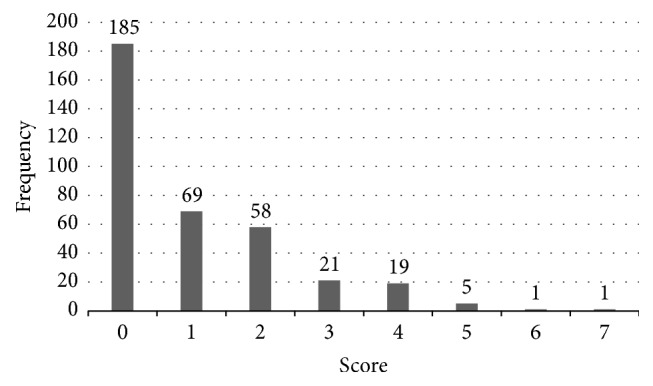
DSM-IV nondepressed individuals by the number of symptoms from DSM-IV depression criteria.

From the 359 individuals who did not meet the DSM-IV criteria for depression, 185 (51.5%) reported not having any of the nine symptoms.  Figure 1 shows the different frequencies of nondepressed individuals with the number of symptoms from the DSM-IV criteria. Only 7 out of the 359 individuals (1.9%) reported having 5 or more symptoms, possibly indicating that they may be at risk of developing depression in the future.

## 4. Discussion

Using the DSM-IV criteria for diagnosing depression, 6.5% (25 out of 384) of the study population had depression. This compares to the estimated prevalence worldwide of 5–10% in the general population. However, the prevalence of depression in this study was slightly higher than the prevalence of common mental disorder found in another study done in the general population in Tanzania [7], but it was much lower than the prevalence of common mental disorders (CMD) in a separate study in Tanzania [6]. The reason for this is probably due to the inclusion of health seeking patients in the later study, as opposed to the general population.

Psychologically traumatized individuals were six times more likely to have depression. This result is in accordance with studies done in Australia [9], Mexico [10], and rural Tanzania [11]. Psychological trauma and therefore psychological stress affect a person's thinking. There is constant negative and pessimistic thinking which affects a person's overall personality and mood, making it bad and therefore meeting the diagnostic criteria of depression. The common psychological stresses that were reported by the study participants were family related, especially problems with in-laws, which was reported by both males and females. Work stress was also an important cause of psychological stress in the individuals. The study was not powered enough to look at gender differences in relation to the different psychological stressors.

Individuals with a positive family history of depression were 2.5 times more likely to have depression. This supports the notion that genetic factors may contribute to depression in individuals.

Individuals with a sufficient family income were five times less likely to get depression. A low income was associated with higher depression rates in studies done in Pakistan [12] and India [13] and among university students in a large-scale study covering 23 countries [14]. A low income is associated with persistent worries about the future and leads to psychological stress and negative thinking.

Female sex (*p* = 0.08) and smoking (*p* = 0.088) may also have an effect on the outcome of depression in an individual; however, the association did not reach statistical significance. Smoking was significantly associated with depression in a study done in Copenhagen [15], but that was not the case in Japan [16]. A bigger sample may give better results for categorically defining the role of gender and smoking in depression.

Based on severity, depressed individuals were 11 times more likely to have recurrent thoughts of death and/or suicide (*p* < 0.05). This difference confirms the fear that severely depressed individuals may need to be followed up more closely and treated adequately to minimize or prevent the incidence of suicide among these individuals.

Efforts should be made to resolve issues which result in psychological stress particularly family issues related to in-laws. Work stress also contributed to psychological stress in the study population and therefore these two aspects can be tackled to reduce the prevalence of depression in the community.

Financial factors are important to take care of to reduce the prevalence of depression in the community. Helping individuals get better jobs or other sources of income to run their families more smoothly will help reduce the financial problems in the community.

The limitation of this study is that the research was conducted during the community events where, in an obligatory manner, attendance is almost over 90%, and therefore representative. However, depressed individuals may still be refraining from attending such a gathering, and hence some individuals may have been missed along these lines.

## 5. Conclusions

This population-based study in the Asian migrant community found that depression was prevalent in 6.5% of the study population. History of psychological trauma, a positive family history of depression, and a low income were significant risk factors for depression. Recurrent thoughts of death/suicide were more common in the depressed group.

## Figures and Tables

**Table 1:** Demographic characteristics of the study population.

Characteristic	Frequency (*N* = 384)
*Age group (in years)*	
Under 25	100 (26.0%)
25 to 34	73 (19.0%)
35 to 44	60 (15.6%)
Over 44	151 (39.3%)
*Sex*	
Female	166 (43.2%)
Male	218 (56.8%)
*Education level*	
Primary education or less	32 (8.3%)
Secondary education	168 (43.8%)
Higher education	184 (47.9%)
*Marital status*	
Single	101 (26.3%)
Married	270 (70.3%)
Divorced	4 (1.0%)
Widowed	9 (2.3%)

**Table 2:** Risk factors against DSM-IV depression status.

Risk factor	Depression		*p* value	OR (95% CI)
	Yes (*n* = 25)	No (*n* = 359)		
*Age group (in years)*				
Under 25	6 (24.0%)	94 (26.2%)		
25 to 34	8 (32.0%)	65 (18.1%)		
35 to 44	2 (8.0%)	58 (16.2%)		
Over 44	9 (36.0%)	142 (39.6%)	0.322	
Sex, female/male (female%)	15/10 (60.0%)	151/208 (42.1%)	**0.08**	**2.07 (0.84–5.15)**
*Education level*				
Primary or less	1 (4.0%)	31 (8.7%)		
Secondary	13 (52.0%)	155 (43.2%)		
Higher	11 (44.0%)	173 (48.2%)	0.768	
*Marital status*				
Single	7 (28.0%)	94 (26.2%)		
Married	16 (64.0%)	254 (70.8%)		
Divorced	1 (4.0%)	2 (0.8%)		
Widowed	1 (4.0%)	8 (2.2%)	0.433	
Presence of comorbidity	11 (44.0%)	106 (29.5%)	0.129	1.88 (0.76–4.60)
Family history of depression	10 (40.0%)	74 (20.6%)	**0.023**	**2.57 (1.02–6.42)**
Smoking	7 (28.0%)	55 (15.3%)	**0.088**	**2.15 (0.77–5.83)**
Previous psychological trauma	10 (40.0%)	34 (9.5%)	**<0.001**	**6.37 (2.42–16.69)**
Insufficient family income (*N* = 266)	5/17 (29.4%)	20/249 (8.0%)	**0.013**	**4.77 (1.30–16.85)**
*Exercise hours per week*				
Less than 2	18 (72.0%)	219 (61.0%)		
2 to 4	5 (20.0%)	86 (24.0%)		
More than 4	2 (8.0%)	54 (15.0%)	0.495	

## Data Availability

The data that support the findings of this study are available from the Muhimbili University of Health and Allied Sciences, but restrictions apply to the availability of these data, which were used under license for the current study and so are not publicly available. Data are however available from the authors upon reasonable request and with permission of Muhimbili University of Health and Allied Sciences.
